# New Weapons to Fight Old Enemies: Novel Strategies for the (Bio)control of Bacterial Biofilms in the Food Industry

**DOI:** 10.3389/fmicb.2016.01641

**Published:** 2016-10-18

**Authors:** Laura M. Coughlan, Paul D. Cotter, Colin Hill, Avelino Alvarez-Ordóñez

**Affiliations:** ^1^Teagasc Food Research CentreCork, Ireland; ^2^School of Microbiology, University College CorkCork, Ireland; ^3^APC Microbiome InstituteCork, Ireland

**Keywords:** biofilm, food, industry, quorum sensing, quorum sensing inhibitors

## Abstract

Biofilms are microbial communities characterized by their adhesion to solid surfaces and the production of a matrix of exopolymeric substances, consisting of polysaccharides, proteins, DNA and lipids, which surround the microorganisms lending structural integrity and a unique biochemical profile to the biofilm. Biofilm formation enhances the ability of the producer/s to persist in a given environment. Pathogenic and spoilage bacterial species capable of forming biofilms are a significant problem for the healthcare and food industries, as their biofilm-forming ability protects them from common cleaning processes and allows them to remain in the environment post-sanitation. In the food industry, persistent bacteria colonize the inside of mixing tanks, vats and tubing, compromising food safety and quality. Strategies to overcome bacterial persistence through inhibition of biofilm formation or removal of mature biofilms are therefore necessary. Current biofilm control strategies employed in the food industry (cleaning and disinfection, material selection and surface preconditioning, plasma treatment, ultrasonication, etc.), although effective to a certain point, fall short of biofilm control. Efforts have been explored, mainly with a view to their application in pharmaceutical and healthcare settings, which focus on targeting molecular determinants regulating biofilm formation. Their application to the food industry would greatly aid efforts to eradicate undesirable bacteria from food processing environments and, ultimately, from food products. These approaches, in contrast to bactericidal approaches, exert less selective pressure which in turn would reduce the likelihood of resistance development. A particularly interesting strategy targets quorum sensing systems, which regulate gene expression in response to fluctuations in cell-population density governing essential cellular processes including biofilm formation. This review article discusses the problems associated with bacterial biofilms in the food industry and summarizes the recent strategies explored to inhibit biofilm formation, with special focus on those targeting quorum sensing.

## Introduction

Certain bacteria develop a fortress or biofilm in the environments they colonize which provides shelter from antimicrobials and other sanitation procedures. A biofilm is formed when planktonic (or free/stand-alone) cells in an aqueous environment adopt a multicellular lifestyle by attachment to, and colonization of, a solid surface (Claessen et al., 2014). This may occur on a submerged surface or at the air-liquid interface (known as pellicle formation; Wu et al., 2012). Some bacteria begin biofilm formation without surface attachment *via* the aggregation of planktonic cells. Subsequent attachment of pre-formed aggregates to a solid surface results in true biofilm formation (Melaugh et al., 2016). The production of an extracellular matrix of DNA, carbohydrates, protein and lipids reinforces the sessile colony, facilitating the trapping of nutrients and protecting it against sanitation and even manual removal.

Biofilm formation is a serious problem in both the food and healthcare industries. Spoilage and pathogenic bacteria colonize, in the form of biofilms, the inside of mixing tanks, vats and tubing, compromising food safety and quality. In hospital settings, biofilm-forming bacteria persist in catheters, implants and on living tissues of patients suffering from chronic infections, such as those caused by *Staphylococcus epidermis* and *Pseudomonas aeruginosa* (Stewart and William Costerton, 2001). Despite the knowledge that the vast majority (~80%) of infectious and persistent bacteria are biofilm-formers (National Institutes of Health, 2002) and that in nature microorganisms are actually forming biofilms (Hall-Stoodley et al., 2004), most of the research carried out to date is focused on the properties and control of planktonic bacteria. In this literature review the knowledge available with respect to biofilm formation in the food industry and current biofilm control strategies is compiled and critically discussed with key focus on anti-biofilm approaches targeting the bacterial quorum sensing system.

## Bacterial Biofilms in the Food Industry

In the food processing industry, microorganisms indigenous to certain foods generally do not harm the consumer and in some cases convey some benefit (e.g., fermented foods in which bacteria are intentionally introduced in the form of a starter culture). Therefore, efforts are not usually made to rid the processing environment of such microbes unless overgrowth or visible product spoilage occurs. Biofilms formed by pathogenic and spoilage microorganisms, however, serve as a reservoir of problematic microbial cells which may contaminate raw materials and food products during processing, resulting in food spoilage and economical losses for the producers (Winkelströter et al., 2014a). Persistence of unwelcome bacteria in industrial settings has been linked to such capabilities as antimicrobial and disinfectant resistance, tolerance of certain environmental stresses and biofilm formation. Consumers may be affected by reduced shelf life of the contaminated product and possible contraction of foodborne illnesses. Fresh, minimally processed foods are at high risk of bacterial contamination. The produce industry, responsible for providing raw and ready-to-eat fruit, vegetables and derived products, faces repeated contamination of food due to spoilage and pathogenic bacteria forming biofilms on industrial equipment or on the foods themselves (Jahid and Ha, 2012). In the dairy industry, a wide range of thermophilic and psychrophilic bacteria dwell along the different stages of processing and pasteurization. Persistent *Bacillus cereus* spores adhered to industrial surfaces act as a conditioning film promoting the prompt attachment of bacterial cells introduced into the system that would otherwise be removed by methods effective against planktonic cells (Marchand et al., 2012). Other thermophilic bacilli, such as *Geobacillus* spp., can grow at temperatures as high as 65°C and their heat-resistant spores prove problematic for the manufacture of milk powders (Palmer et al., 2010). Psychrotrophic bacteria complicate storage of milk and other dairy products as they can thrive at refrigeration temperatures. *Pseudomonas* are common spoilage psychrophiles which can reach high population numbers and form biofilms at low temperatures on walls of milk cooling tanks and pipelines prior to heat processing and often secrete heat-stable lipolytic and proteolytic enzymes which contribute greatly to milk spoilage (Marchand et al., 2009). In addition, *Pseudomonas* biofilms have been shown to be capable of providing shelter to other pathogenic bacteria (e.g., *Listeria monocytogenes*) in multi-species biofilms (Marchand et al., 2012). *L. monocytogenes* is an important psychrotrophic food pathogen associated with the dairy (as well as the produce and poultry) industry. It is an opportunistic gastrointestinal (GI) foodborne pathogen also capable of causing serious systemic infectious disease (listeriosis) in certain individuals including the very young, the elderly, in pregnant woman and immunocompromised patients (Hamon et al., 2006; Freitag et al., 2009). The seriousness of *L. monocytogenes* occupancy in food related environments and, subsequently, the human host is as a result of the bacteria’s ability to multiply at a wide range of temperatures (Walker et al., 1990) and to tolerate and adapt to harsh environmental conditions such as osmotic stress (Dykes and Moorhead, 2000) and bile acid in the human GI tract (Gahan and Hill, 2014). This resistance to harsh conditions and its ability to form biofilms allow *L. monocytogenes* to persist in food processing environments, a serious threat to the food industry. Indeed, the persistence of several specific *L. monocytogenes* strains in food and food processing areas across seven out of 48 processing facilities in the Republic of Ireland over a period of 12 months has recently been demonstrated (Leong et al., 2014). Infections caused by food-associated pathogens capable of forming biofilms, e.g., *L. monocytogenes, Campylobacter* spp., *Salmonella* spp., seriously impact public health on a global scale with the annual health-care costs associated with common food-borne pathogens reaching $15.5 billion in the USA per year (EFSA, 2009; Scallan et al., 2011; Hoffmann et al., 2015). Infection with *Campylobacter* species is the leading cause of food-borne bacterial gastroenteritis worldwide (World Health Organization, 2012) with *Campylobacter jejuni* claiming responsibility for the majority of those cases. Acute infection may lead to serious complications with long term consequences such as peripheral neuropathy symptoms typical of Guillain–Barre syndrome (GBS) which has long been associated with *Campylobacter* infection (Nachamkin et al., 1998), reactive arthritis (Pope et al., 2007) and post-infectious irritable bowel syndrome (IBS; Schwille-Kiuntke et al., 2011). *C. jejuni* readily forms biofilm on food industry related surfaces (Teh et al., 2014), is frequently associated with poultry, and it has even been demonstrated that chicken juice increases biofilm formation on food industry-related equipment (Brown et al., 2014). Another serious pathogen is *Salmonella enterica* serovar Typhi (*S.* Typhi), the causative agent of typhoid fever, which is responsible for 21.7 million human infections and 217,000 deaths annually (Crump and Mintz, 2010) and is capable of forming biofilms (Kalai Chelvam et al., 2014) and persisting on materials often used in the food industry such as stainless steel, rubber and plastics, as comprehensively reviewed by Steenackers et al. (2012). Additionally, other *Salmonella* serovars able to form biofilm on food-related surfaces, such as *S. enterica* serovar Typhimurium (*S.* Typhimurium), cause a typhoid-like disease which is usually not fatal to healthy individuals but is commonly the source of poultry and meat products-related food poisoning (Jackson et al., 2013).

## Biofilm Formation and Regulation

**FIGURE 1:**
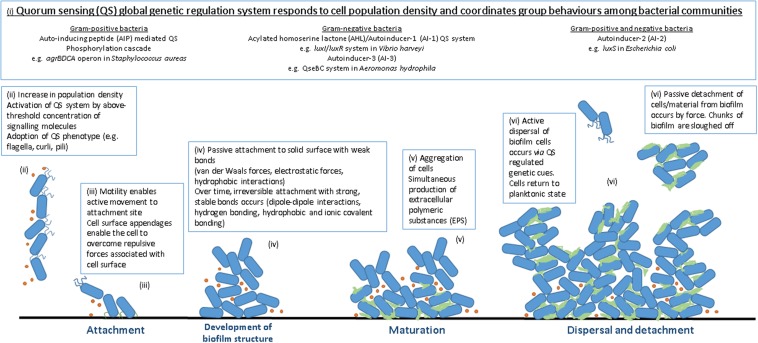
**Stages of biofilm formation.** (i) QS signaling molecules (ii) high population density, high QS signal (iii) attachment to solid surface (iv) increase in cell numbers, irreversible attachment, development of biofilm structure (v) biofilm maturation and EPS production (vi) dispersal.

Biofilm formation occurs over a series of sequential steps, in short: attachment (reversible and irreversible), cell-to-cell adhesion, expansion, maturation, and dispersal (**Figure 1**). Successful attachment to solid surfaces is governed by a slew of factors concerning both the bacterial cell and the surface of the potential biofilm site (reviewed by Chmielewski and Frank, 2003; Persat et al., 2015). Biofilm-forming bacteria possess motility and anchoring appendages which enable movement through liquid and attachment to an appropriate surface such as flagella are proteinaceous structures protruding from the bacterial cell surface which enable swimming motility (Van Houdt and Michiels, 2010). Other adhesion molecules such as pili (or fimbriae) (Mandlik et al., 2008) and curli (Cookson et al., 2002) contribute to biofilm formation by enabling active attachment. Once attached, the bacteria proceed to colonize the surface through the formation of cellular aggregates known as microcolonies. Under permissive environmental conditions, microcolonies form two-dimensional dynamic structures as cell numbers increase, the first step toward structural organization on the chosen surface. This framework further matures into a defined architecture with cells arranged in simple or elaborate structures suited to thriving in their particular environment (Pilchová et al., 2014). Mature biofilm formations include flat monolayers, three-dimensional structures or mushroom- or tulip-like assemblies with low surface coverage and intervening water channels for nutrient and waste exchange (Karatan and Watnick, 2009; Jahid and Ha, 2012). Exopolymeric substance (EPS) is a gelatinous material encasing the cells of a biofilm which is composed of substances excreted by the cells themselves including proteins, polysaccharides, nucleic acids, lipids, dead bacterial cells, and other polymeric substances hydrated to 85–95% water (Costerton et al., 1981; Sutherland, 1983). EPS functions to anchor to biotic and abiotic surfaces (Characklis and Marshall, 1990), concentrate nutrients from the surrounding environment within the biofilm, limit access of antimicrobial agents (contributing to resistance) and prevent the biofilm from desiccation (Carpentier and Cerf, 1993). The final stage in the biofilm life cycle involves the return of a number of adhered cells to the surrounding environment. In active detachment cells revert back to their planktonic state and leave the biofilm in response to cellular cues (encouraging them to search for an additional attachment site when conditions are favorable). Passive detachment occurs as a result of environmental changes, such as nutrient availability and movement of surrounding liquid, and involves the sloughing off or erosion of parts of the biofilm by chemical means or force (Kaplan, 2010). Dispersal (reviewed by McDougald et al., 2012) facilitates the spreading of bacterial contaminants and the spoilage of foodstuffs by allowing the biofilm to act as a reservoir releasing cells back into the environment to carry out the cycle elsewhere.

Biofilms in nature and, indeed, in the food industry generally consist of multiple bacterial species as opposed to the mono-species biofilms usually cultured in laboratory studies (Yang et al., 2011). Life in a multispecies biofilm is advantageous, providing increased shelter and resistance to antimicrobials compared to corresponding single species biofilms (Burmølle et al., 2006). A study by van der Veen and Abee (2011) demonstrated that mixed species biofilms containing two *L. monocytogenes* strains and a *Lactobacillus plantarum* strain displayed increased resistance to the commonly used disinfectants benzalkonium chloride and peracetic acid in comparison to disinfection carried out on monospecies biofilms formed by the same strains. Wang et al. (2013) investigated the biocidal effect of the commercial sanitizer Vanquish (a quaternary ammonium compound-based product) and a chlorine solution prepared from Clorox (a germicidal bleach product) on mono- and multispecies biofilms formed by several Shiga toxin-producing *Escherichia coli* O157:H7 and *S. enterica* serovar Typhimurium strains. Increased resistance to sanitizers was observed in multispecies biofilms in which one of the strains was an EPS producer. EPS-producing strains of one species conferred protection to non EPS-producing strains of another, ultimately protecting both (to some degree) from sanitation. The results suggest the importance of the EPS component of bacterial biofilms in conveying resistance to the producer and in this case to the companion strains of mixed biofilms.

Cells that form biofilms have unique properties that enable them to do so, the expression of which is under the control of a global gene regulation system that responds to fluctuations in population density, known as quorum sensing (QS; Fuqua et al., 1994). Specific signaling molecules are produced and detected, governing community behavior. The higher the population density is, the higher the concentration of signaling peptides reached. When a minimal threshold stimulatory concentration of signaling molecules is reached, the QS system is activated and, thus, expression of QS-related genes occurs. QS is responsible for organizing the expression of many genes including those involved in essential cell processes, those encoding various virulence factors and also genes regulating biofilm formation. QS may be organized into three main sub-systems, classified by the type of signaling molecules employed: the acyl homoserine lactone (AHL) or autoinducer-I (AI-I) system is observed in Gram-negative bacteria, the peptide-mediated QS system in Gram-positive bacteria and the autoinducer-2 (AI-2) system present in both Gram-negative and Gram-positive bacteria.

Acyl homoserine lactones were originally discovered in marine bacteria (*Vibrio* spp.) having been found to be responsible for bioluminescence regulation, and have since been identified in numerous Gram-negative bacteria. Synthesis of an AHL signaling molecule involving a LuxI type protein occurs when an acyl-carrier protein-bound fatty acyl derivative is transferred to the amino group of *S*-adenosyl-methionine (SAM; Brackman and Coenye, 2015). AHL-mediated QS is well-described by Waters and Bassler (2005) using the control of the *Vibrio fischeri* luciferase operon as an example. Different bacteria produce different types of AHLs, controlling a range of functions. In addition, the same AHL may be produced by a number of bacteria spanning several genera. All AHLs contain the same homoserine lactone moiety but differ in the length and structure of their acyl groups. The diversity and specificity of AHL molecules, conveyed by the length, backbone and saturation of their fatty acyl side chains suggests their function in intraspecies communication. These N-acylated side chains vary in length from 4 (e.g., C4-HSL) to 18 carbons often with an oxo (e.g., 3-oxo-C6-HSL) or hydroxyl group (e.g., 3-hydroxyl-C6-HSL) on their third carbon atom and may also contain double bonds (Skandamis and Nychas, 2012). A huge variety of AHLs exists and has been reported in a wide range of bacterial species, including microorganisms associated with food and food processing. For example the common milk contaminant *Pseudomonas fluorescens* produces both C4-HSL and 3-oxo-C8-HSL AHL signaling molecules (Liu et al., 2007). *Hafnia alvei*, which is often isolated from cheese, produces the AHL *N*-3-oxohexanoyl HSL (Bruhn et al., 2004). In fact, AHL production by food-dwelling species has been associated with food spoilage. The detection of AHLs in some spoiled foods has led to suggestions that the secretion of certain proteolytic, saccharolytic and lipolytic enzymes, associated with food spoilage, is under the influence of AHL signaling (reviewed by Bai and Rai, 2011; Skandamis and Nychas, 2012).

It has been proposed that the AI-2 signaling system is used for both inter and intraspecies bacterial communication as AI-2 signaling molecules are non-specific. This system was first identified in *Vibrio harveyi*, an AHL-deficient strain which was capable of producing the bacterium’s characteristic bioluminescence suggesting that another regulatory system was responsible for controlling its operation (Bassler et al., 1993). AI-2 synthesis involves two major enzymatic steps (Brackman and Coenye, 2015): 5′ methylthioadenosine nucleosidase (MTAN which is encoded by *pfs*) is produced and cleaves adenine from *S*-adenosyl-homocysteine (SAH). This results in the production of *S*-ribosyl-homocysteine (SRH), which is subsequently cleaved by LuxS to form 4, 5-dihydroxy-2, 3-pentanedione (DPD). Spontaneous rearrangements and modifications of DPD yield a combination of molecules collectively referred to as AI-2. The presence of *luxS*, and thus AI-2 mediated QS, has been reported in some foodborne pathogens. Reeser et al. (2007) showed that AI-2 is critical for mature biofilm formation in *C. jejuni* M129 through the construction of a *luxS* deficient mutant. This strain, unable to produce the QS signaling molecule AI-2, was seen to have greatly decreased biofilm formation at the 48 and 72 h time points when compared to the wild type of the same strain, despite both having a similar growth rate. The group also showed that flagella are important for biofilm formation to the strain at hand by constructing a *flaAb* mutant, which also showed reduced biofilm formation at the 48 and 72 h time points and again no changes in growth rate. AI-2-like activity has also been reported in *L. monocytogenes* and deletion of the *luxS* gene resulted in the bacterium forming thicker than normal biofilm, indicating a strong link between AI-2 signaling and biofilm regulation in *L. monocytogenes* (Sela et al., 2006). More recently, the relationship between *luxS* and biofilm formation was demonstrated in *E. coli* by Niu et al. (2013) by comparing the biofilm forming abilities of a modified set of *E. coli* W3110 (a laboratory strain) with the wild type. The set included a *luxS* deficient mutant, a *luxS* mutant carrying an inducible plasmid containing *luxS* complement and a *luxS* mutant hosting a blank pBAD18 plasmid as a negative control. AI-2 production, quantified by measuring bioluminescence induced in the reporter strain *V. harveyi* BB170, was observed to be higher in the *luxS* complement strain than the wild type and absent in both the *luxS* mutant and the negative control. Following on from this, biofilm formation in a continuous flow cell was assessed by differential interference contrast (DIC) light microscopy and confocal laser scanning microscopy (CLSM) for each strain. While the *luxS* mutant and the negative control were found to form compact clusters, the *luxS* complement formed tall, thick biofilms and the wild type a combination of the observed phenotypes. The results indicate a strong correlation between AI-2 expression and quality of biofilm, suggesting the key role of AI-2 mediated QS in biofilm formation in *E. coli* W3110. As well as the Gram-negative microbes mentioned above, *luxS* has also been studied in Gram-positive bacteria. *Bacillus subtilis*, a spoilage bacterium regularly isolated from dairy products and processing facilities (reviewed by Gopal et al., 2015) was reported to regulate biofilm formation through *luxS*-mediated quorum sensing (Duanis-Assaf et al., 2015).

The presence of a third autoinducing molecule (AI-3) has been reported in Gram-negative bacteria such as *E. coli, Klebsiella pneumoniae*, *Shigella* spp., *Salmonella* spp., and *Enterobacter cloacae* (Walters et al., 2006). Sperandio et al. (2003) first described AI-3 when studying gene expression of the foodborne pathogen *E. coli* O157:H7 in response to a eukaryotic cell signal. The group found AI-3 (presumed to be LuxS-dependent) to be responsible for the activation of virulence gene expression, including flagella regulation genes, and proposed AI-3 as a possible agent of cross-communication between bacterial and host cells as substitution of either AI-3 or the mammalian hormone epinephrine (Epi) restored the virulence phenotype in a *luxS* deficient mutant, suggesting that AI-3 and Epi employ the same signaling pathway. A later study by Walters et al. (2006) showed that *luxS* mutants were forced to synthesize homocysteine *via* an alternative pathway using oxaloacetate and that culturing the mutants in media supplemented with L-aspartate alleviated the demand for oxaloacetate and restored AI-3 production without affecting AI-2 production. This work demonstrates that AI-3 production is not LuxS-dependent and the true mechanism for synthesis of this molecule is yet unclear (reviewed by Bai and Rai, 2011).

In Gram-positive bacteria, QS communication is mediated by autoinducing peptides (AIPs; Bai and Rai, 2011). Bacteria employing this system do so with unique, species-specific signaling molecules, suggesting that peptide-mediated signaling enables intraspecies communication alone. The biphasic mode of infection employed by *Staphylococcus aureus* is an elegant example of QS signaling in Gram-positive bacteria, reviewed by Waters and Bassler (2005). Examples of bacteria employing QS peptide signaling are the opportunistic foodborne pathogen *Clostridium perfringens*, for the regulation of virulence, sporulation, toxin production (Ma et al., 2015) and biofilm formation (Vidal et al., 2015), and *L. monocytogenes* for virulence, invasion and biofilm regulation (Riedel et al., 2009; Abee et al., 2011).

## Strategies Undertaken to Prevent Biofilm Formation and Remove Existing Biofilms

The best strategy to eradicate bacterial biofilms from food-related environments is to prevent their formation. This can be achieved by preventing the presence of biofilm forming bacteria in critical areas, e.g., sterile manufacture (aseptic processing) or terminal sterilization of parenteral preparations and equipment. In most cases, especially in food production, sterility of the environment is neither possible nor cost-effective and so measures are taken to instead reduce the numbers of harmful and biofilm-forming bacteria in the production area. In food production facilities, detailed hygiene practices are carried out by trained staff in an effort to prevent the introduction of microbes into the processing and finishing areas. Daily sanitation/disinfection processes are carried out in every food manufacturing plant to eliminate microbes that have made it inside and aim to prevent colonization or persistence. The measures involved incorporate mechanical, chemical, and thermal processes to prevent biofilm formation as efficiently as possible.

### Cleaning and Disinfection

Measures such as Good Manufacturing Practice (GMP) and Hazard Analysis Critical Control Point (HACCP) schemes (Sharma and Anand, 2002) are active in food processing facilities to ensure that food quality and safety meet high standards. Documented and validated cleaning procedures exist and their implementation is legally enforced *via* inspection by regulatory bodies. A general cleaning procedure for food processing and production areas involves six necessary sequential steps: pre-clean (physical), washing (detergents), rinsing, sanitation, final rinsing, and drying (Safefood, 2012). The first of these is a preparatory measure known as a gross (or dry) clean, the aim of which is to manually remove all bulk soil, packaging materials and tools, essentially all unnecessary equipment and large debris. Equipment to be manually cleaned must also be disassembled and laid out for ease of access during the subsequent steps. In dairy manufacturing plants (DMPs), and others, a control protocol known as Clean-In-Place (CIP) is implemented to reduce biofilm formation and microbial load in general (Bremer et al., 2006). CIP is a semi- or fully automated programmed cycle of timed rinsing and cleaning stages for the efficient cleaning of equipment interiors that are inaccessible or their manual cleaning ineffectual. Next, a pre-rinse is carried out during which the equipment and area is rinsed with water until surfaces are visibly clear of soils and deposits. Higher water pressure may be used for removal of stubborn soils though care must be taken not to cause cross-contamination through splash-back or migration of aerosolized water onto other surfaces. Following this step, excess water must be removed to avoid pooling around or backing up of drains and to prevent dilution of the cleaning solutions/solvents used in later steps. The next step involves the application of a detergent to remove remaining food deposits such as proteins and grease, layers in which bacteria can survive and re-enter the system post-cleaning. Detergents may be applied in the form of foam or aerosol spray, at an appropriate concentration, and adequate contact time with surfaces must be allowed to ensure efficient action. Alkaline and acidic products are commonly used detergents in the food industry (Simões et al., 2010) with alkalis showing success in the removal of *Pseudomonas putida* biofilms from stainless steel (Antoniouand and Frank, 2005). In the following step of the cleaning protocol, detergent and lifted food deposits are removed from the area through rinsing with water at the lowest effective pressure. The surfaces should be visibly clean and free of layers of soil and any marks or residues left by the detergent. Again, excess water is evacuated. At this stage, disinfection is performed to reduce microbial load. Disinfectants may be applied as a liquid spray directly to surfaces or as a fine mist *via* aerial fogging to target airborne microorganisms, which then also settles on and disinfects surfaces. The ambient temperature and the contact time between the disinfectant solution and the surface should be factored into the procedure to maximize the biocidal effect. Some commonly used disinfectants that have demonstrated competence in reducing biofilms in the food industry include hydrogen peroxide (H_2_O_2_), sodium hypochlorite (NaClO), which is also an effective sanitizer, ozone, and peracetic acid (Srey et al., 2013). Toté et al. (2010) found H_2_O_2_ and NaClO to be effective in the removal of *S. aureus* and *P. aeruginosa* biofilm cells and EPS matrix from 96-well assay plates. It has been demonstrated that ozone and especially H_2_O_2_ are effective at inhibiting *Vibrio* spp. biofilms associated with seawater distribution networks used in fish-processing plants (Shikongo-Nambabi et al., 2010) and also that peracetic acid is active against *L. monocytogenes* biofilms (Cabeça et al., 2012). Although sanitizers, which possess the combined action of both detergents and disinfectants, are used in some cleaning protocols, it is believed that splitting these steps and introducing an intermediate rinsing step is more effective than sanitizing alone. Even so, sanitizers remain in use and sanitizing compounds such as NaClO and Spartec, a quaternary ammonium compound (QAC), have been found to be effective against *B. cereus* biofilms when applied under specific cleaning protocols (Peng et al., 2002). The next stage in the cleaning process is the rinsing away of the disinfectant. Most disinfectants are safe to leave on surfaces that do not have direct contact with food, however water of a high quality is used to rinse food contact surfaces and in some cases non-contact surfaces as well. Finally, the equipment is dried to remove rinsing water. Although regular application of cleaning agents reduces microbial populations (Jahid and Ha, 2012), it is normally not efficient at removing mature biofilms. Cleaning and disinfection can remove unwanted bacteria before they have a chance to attach to a surface and form a biofilm, however, due to the fast rate at which attachment and biofilm formation occurs, they are not completely efficient at preventing contamination of food processing environments. In addition, due to residual soil and previous biofilm matrix present on surfaces, sanitation may not be effective alone and the use of disinfectants may select for resistant bacteria (Simões et al., 2010). Interestingly, bacteria residing in biofilm matrices are remarkably (100–1000 times) more resistant to cleaning and sanitation processes than planktonic cells (Gilbert et al., 2002) and it is noteworthy that the majority of chemical disinfectants that are commonly implemented in food, industrial, clinical and domestic cleaning procedures are based on bactericidal studies performed on planktonic cells (Annonymous, 1997). The reasons for increased resistance of bacteria in biofilms are not yet fully understood but the phenomenon has been well-documented (Nickel et al., 1985; Luppens et al., 2002).

### Processing Equipment Materials and Design

Facility design and staff training is highly important for minimizing cross-contamination between high risk and low-risk areas within the plant that can be caused by unchecked foot traffic between stations. Zone establishment segregating exposed product areas from packaging areas, the limiting of access to high-risk areas to authorized personnel and strict garbing and hand-washing requirements on entering restricted areas all play a role in maintaining hygiene standards. Cross departmental knowledge and awareness of potential consequences of contamination ensures compliance and lessens the likelihood of accidental breach of policy. Included in facility design is the selection of appropriate materials for use in the processing areas. Materials for the design of food processing and manufacturing equipment are selected based on a number of factors, most importantly ease of cleaning for reduction of contamination and associated risks. Materials should also be reasonably resistant to chemical and age-related corrosion for maintenance of a smooth and easy-to-clean surface and to prevent contamination risks and downtime associated with frequent replacement of damaged/corroded equipment. Surface topography is important as microorganisms may attach or find shelter in cracks, scratches, and corners of equipment making them extremely difficult to remove (Bremer et al., 2006). Inert metals are commonly used in the food industry, especially stainless steel and aluminum. Stainless steels contain alloys such as chromium to increase resistance to corrosion (rusting). Type 316 steel is especially resistant to chloride environments and is more costly than type 304 steel which is more commonly used due to its versatility and ease of forming. The smooth surface finishes that are achieved by rolling and polishing steel make it a very valuable material for the production of food processing equipment. Another commonly employed metal is aluminum, a light weight and economical material which is also highly resistant to corrosion, especially from acids. Aluminum, however, is susceptible to scratching and damage due to a low surface hardness and to corrosion by alkalis, traits which allow the smooth surface to be compromised, increasing the risk of contamination. In milk processing facilities equipment is required to be resistant to corrosion in alkaline and/or acidic conditions (Marchand et al., 2012) and so stainless steel is normally used. Non-metal materials are employed for moving and disposable equipment such as conveyor belts, containers and cutting boards and for components and attachments where soft material is required such as for seals, gaskets, membranes, and piping. These materials are most commonly elastomers (rubbers) such as ethylene propylene diene monomer rubber (EPDM), nitril butyl rubber (NBR, aka Buna-N^®^), silicon rubber or fluoroelastomer (Viton) and plastics such as polypropylene (PP), polycarbonate (PC), high-density polyethylene (HDPE), unplasticized polyvinyl chloride (PVC), and fluoropolymers such as polytetrafluoroethylene (PTFE aka Teflon^®^; Faille and Carpentier, 2009; Marchand et al., 2012). Unfortunately, certain bacteria are capable of forming biofilms on these food-approved materials. This attachment is aided by improper cleaning of such materials as soil or debris remaining post-sanitation may form a conditioning film for subsequent attachment of planktonic bacteria to this site (Marchand et al., 2012). Surface preconditioning using surfactants that modify the chemical properties of surfaces have been used to prevent bacteria from attaching (Simões et al., 2010). Indeed, more than 90% inhibition of *P. aeruginosa* adhesion to stainless steel and glass was reported by Cloete and Jacobs (2001) upon treating the surfaces with ionic and anionic surfactants. Biosurfactants, microbial compounds that act as surfactants, may also be employed to reduce or prevent adhesion of problematic biofilm-forming bacteria (Banat et al., 2010). Zezzi do Valle Gomes and Nitschke (2012) investigated the efficacy of biosurfactants such as surfactin from *B. subtilis* and rhamnolipids from *P. aeruginosa* in reducing the adhesion and disrupting the pre-formed biofilms of the pathogenic food-associated bacteria *L. monocytogenes*, *S. aureus*, and *Salmonella* Enteritidis. The biosurfactants studied were effective in the disruption of biofilms formed on polystyrene microplates by all species individually and in the disruption of a multispecies biofilm containing all three. The action of the surfactants in preventing bacterial adhesion was effective against pure culture biofilms of each species. However, they were shown to have reduced impact in preventing adhesion of the mixed bacterial culture to the plates, again highlighting the advantages bestowed to bacteria residing in a multispecies habitat. Gu et al. (2016) reported the effective removal of established *P. aeruginosa* PAO1, *S. aureus* ALC2085 and uropathogenic *E. coli* ATCC53505 biofilms formed on an antifouling surface. The group used shape memory polymers (SMPs) -a type of material specially designed to remember a particular shape, manipulated into keeping a temporary shape and then coaxed back into its original form by external activation- as an attachment surface for the microbes to form biofilm and, upon triggering of SMP shape change, the amounts of adhered cells were dramatically reduced (99.9% in the case of *P. aeruginosa*). This type of study takes anti-biofilm surface topography research to a new level, achieving the physical displacement of established biofilms with minimal (if any) effect on the surrounding environment using biocompatible materials and may in time be applicable to equipment and facility design in the food industry.

### Processing Conditions

Another approach to prevent biofilm formation of bacteria present in the production environment involves carrying out the process under conditions unfavorable to biofilm formation. Temperature appears to influence bacterial attachment to solid surfaces. Cappello and Guglielmino (2006) studied the adhesion of *P. aeruginosa* ATCC 27853 to polystyrene plates at 15, 30, and 47°C, reporting a dramatic difference in adhesive ability (measured as percentage hydrophobicity) between cells cultured at the higher temperatures of 30 and 47°C and cells cultured at 15°C. Temperature-dependent variation in biofilm formation was also observed among *L. monocytogenes* strains by Di Bonaventura et al. (2008). In addition to temperature, nutrient availability in a given environment has been shown to influence the quality of biofilms formed. In general, studies have demonstrated that biofilms formed under low nutrient availability or starvation conditions are superior to biofilms formed under high nutrient availability, with bacteria in nutrient rich surroundings failing to form biofilms in some cases (Petrova and Sauer, 2012). Zhou et al. (2012) reported enhanced (thicker and more complex) biofilm formation of *L. monocytogenes* in a poor minimal essential medium (MEM) supplemented with glucose compared to the biofilm formed by the same strain in nutrient rich brain heart infusion (BHI) broth. Similar results were seen previously by Dewanti and Wong (1995) who cultured *E. coli* 0157:H7 biofilms on stainless steel chips in broths of varying nutrient availability. The group reported the formation of biofilms with high cell numbers that formed quickly and produced thicker EPS when grown in nutrient-scarce media in comparison to those formed in tryptic soy broth (TSB). Biofilm formation may also be altered by the pH of the surrounding media. Decreased cell attachment was reported (Tresse et al., 2006) for *L. monocytogenes* biofilms grown at pH 5 than for those at pH 7, which was later (Tresse et al., 2009) attributed to pH-dependent flagellation in *L. monocytogenes* observed as a down-regulation of flagellin synthesis in acidic conditions. O’Leary et al. (2015) investigated the effect of low pH on the biofilm forming capacity of four acid-adapted *S.* Typhimurium DT104 strains, only one of which formed biofilms at both pH 5 and 7, with the remaining three strains unable to form stable biofilms at the mildly acidic pH of 5. Gene expression under the distinct pH conditions was also examined showing that genes involved in biofilm formation were expressed at higher levels at pH 5 than at neutral pH for all isolates, despite the lack of biofilm formation observed in three out of four strains. These results propose the existence of a separate set of genes which aid biofilm formation under acidic conditions and which were not present in three of the strains at hand. Despite the successes of biofilm-limiting conditions in laboratory experiments, in most cases, application of these findings to the food industry is not appropriate as altering process conditions is likely to impact product quality.

### Physical Approaches

Physical force is also employed in the food industry for the reduction of microbial load and the removal of biofilms. Brushes, water jets, and turbulent flow in pipelines are used to administer force to susceptible surfaces during cleaning protocols (Safefood, 2012). In addition, in recent years, other physical-based novel technologies have been developed to reduce the microbial load on surfaces or remove biofilms. Plasma treatment involves bombarding surfaces with a partially ionized gas and has been used successfully as a disinfectant targeting planktonic microbes (Laroussi, 1996). A study carried out by Vandervoort and Brelles-Mariño (2014) demonstrated the efficacy of plasma-mediated inactivation against a *P. aeruginosa* biofilm grown on borosilicate glass in continuous culture, better to mimic natural and industrial environmental conditions under which problematic biofilms are generally formed. The group reported changes in biofilm structure post-plasma treatment which they associated with decreased adhesion of the biofilm to the colonized surface. Ultrasonication was found to be successful for the removal of biofilms when used in combination with other anti-biofilm agents such as antibiotics (Peterson and Pitt, 2000), ozone (Baumann et al., 2009) and the chelating agent ethylenediaminetetraacetic acid (EDTA) (Oulahal et al., 2007), reviewed by Srey et al. (2013). A greater understanding of the intricacies of a biofilm (species involved, structure, composition of EPS, etc.) leads to improved, more focused efforts to remove existing biofilms and prevention of biofilm formation of studied species. Manual removal of cells from a biofilm and simple analysis by cell plating followed by microscopic analysis of fluorescently labeled or stained lab-grown biofilms (cultured in high throughput matrices such as 96-well plates or glass/stainless steel coupons) provides detailed information on both the microbes involved and on biofilm architecture. Quantification of live cells (e.g., MTT staining) or biofilm formed (crystal violet staining) may be carried out on cultured biofilms to quantify total biomass, assess external factors and environmental conditions affecting biofilm formation and to evaluate the success of biofilm removal and inhibition strategies, as reviewed by Stiefel et al. (2016). Additionally, polymerase chain reaction (PCR)-based methods allow for rapid detection of pathogens and spoilage bacteria from a biofilm sample, as reviewed by Winkelströter et al. (2014b). Dzieciol et al. (2016) used culture independent methods (pyrosequencing of 16S rRNA gene amplicons) to characterize the microbial communities of floor drain water from four sources in a cheese processing facility for the purpose of monitoring *L. monocytogenes* persistence. Other useful technologies include biofilm detectors which are used to monitor biofilm formation on a surface and can enable intervention in the early stages of biofilm formation in an attempt to prevent its progression into a mature biofilm. Pereira et al. (2008) developed a surface sensor capable of detecting early biofilms, and further developed the technology to monitor cleaning-in-place procedures (Pereira et al., 2009). Al-Adawi et al. (2016) employed CLSM and denaturing gradient gel electrophoresis (DGGE) to study mono and dual species biofilm formation of food-related pathogens on stainless steel and raw chicken meat and the transfer of microbial cells from the abiotic to the biotic surface. As biofilms contribute hugely to cross contamination between equipment in the food industry and the products themselves, such studies are critical in developing novel and appropriate techniques for detecting and analyzing biofilms.

The majority of current strategies aim to prevent introduction of microbes into the food processing environment, contributing also to reduce the risk of biofilm formation through removal of soils and food deposits on processing equipment as improperly cleaned surfaces with soil build-up serve as attachment sites for biofilm forming bacteria. However, most of these approaches do little to remove existing biofilms formed by persistent bacteria within production areas, for example biofilms in milk tanks and tubing that are heat tolerant or thermophilic and are resistant to the high temperatures of pasteurization. Periodic cleaning of equipment requires halting production, drainage and cleaning which negatively impacts output and is not ideal in terms of hygiene.

### Enzymes

**FIGURE 2:**
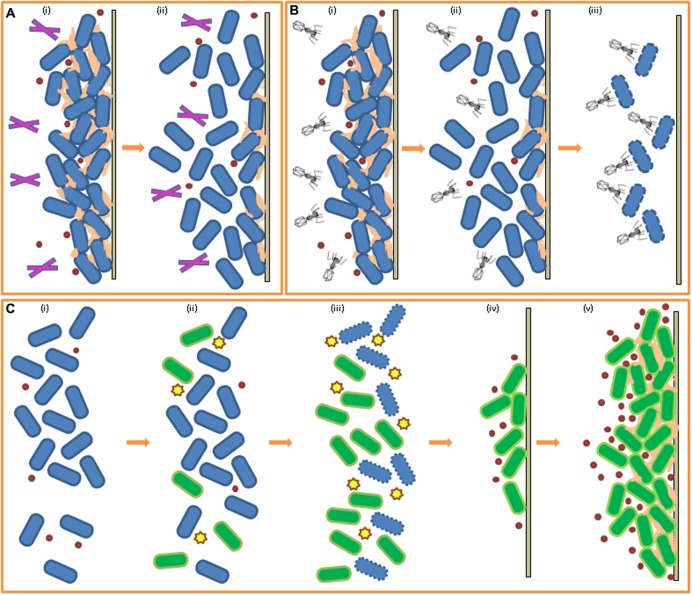
**Biofilm control through enzymes, phage, and bacteriocins. (A)** Effect of enzymes on pre-existing biofilm (i) biofilm formed, EPS production, addition of enzymes (ii) breakdown of EPS and biofilm reduction by enzymatic action. **(B)** Effect of bacteriophage on pre-existing biofilm (i) biofilm formed, EPS production, addition of phage (ii) degradation of EPS by phage, reduction of biofilm (iii) bacterial cells in biofilm targeted by targeted for infection by phage. **(C)** Effect of bacteriocins and competitive exclusion on biofilm-forming cells (i) planktonic cells of species A (blue) (ii) addition of bacteriocin-producing species B (green) (iii) targeting of species A by bacteriocins, increase in number of species B cells (iv) increase in QS molecule concentration for species B, attachment to solid surface (v) biofilm formation of species B in place of species A.

Enzyme-based detergents are used to improve efficacy of disinfectants against bacterial biofilms. Enzymes can target cells in the biofilm matrix and can cause the matrix to become looser and break up. They can also trigger cell release actions in the biofilm enveloped cells, causing an amount of cells to break off from the biofilm. Enzymes have some role in targeting the bacterial cells encased within a biofilm, however the main function of enzymes is to degrade the lipid, carbohydrate and DNA components of the extracellular matrix, severing the links between cells and subsequently separating them, allowing rapid deterioration of the biofilm integrity (see **Figure 2A**). Disinfectants can then act more powerfully to kill cells that were once embedded in the matrix of the biofilm EPS and can also target released cells which have been forced into the planktonic state by the enzymes action. The types of enzymes commonly employed depend on the composition of the biofilm one is attempting to eradicate and include proteases, cellulases, polysaccharide depolymerases, alginate lyases, dispersin B and DNAses (Bridier et al., 2015). As EPS is a heterogenic matrix, a combination of enzymes with different target substrates is used, and even further tweaking of the mixture is required for multispecies biofilms where there exists a variety of substrates. A study by Walker et al. (2007) demonstrated the success of an enzyme mix against a multispecies biofilm formed on brewery dispense equipment. Additional studies have been carried out which highlight the potency of enzyme-based approaches against food related bacterial biofilms. Mimicking a meat processing environment, Wang et al. (2016) induced biofilm formation by a cocktail of seven *Salmonella* spp. strains isolated from meat processing surfaces and poultry grown in meat thawing-loss broth (MTLB) and on stainless steel. They reported the successful removal of said biofilm through treatment with cellulase followed by immersion in cetyltrimethyl ammonium bromide (CTAB). Oulahal-Lagsir et al. (2003) reported a 61–96% removal of *E. coli* biofilms formed on stainless steel in milk when they synergistically exposed the biofilms to both proteolytic and glycolytic enzymes and ultrasonic waves for 10 s. The action of polysaccharidases against *P. fluorescens* biofilms and the efficacy of serine proteases in the removal of *Bacillus* biofilms from stainless steel chips was reported by Lequette et al. (2010). Commercial α-amylases have been found to be effective at both removal and inhibition of *S. aureus* biofilms (Craigen et al., 2011). Another study investigated the potential for commercial proteases and amylases to break down the EPS of biofilms formed by *P. fluorescens* on glass wool (Molobela et al., 2010). The group examined the composition of the EPS and selected appropriate enzymes, which were evaluated as anti-biofilm agents. As the EPS in this case consisted predominantly of proteins, commercial proteases were found to be most effective at biofilm removal in this study. Enzymes sourced from fungal strains were also shown to be successful at removal of biofilms formed by *P. fluorescens* on glass coupons (Orgaz et al., 2006). When employing enzyme-based products, one must consider the reaction of enzymes with food products or ingredients during processing, for example, Augustin et al. (2004) found several commercial enzymes to be useful as cleaning products against biofilms of common dairy-associated spoilage bacterium *P. aeruginosa*. However, the activity of proteinase enzymes is reduced in the presence of milk and so the performance of the enzyme was not sufficient to encourage further development of a product. DNases, which degrade the extracellular DNA component of EPS, have also been studied as enzyme-based formulations for battling biofilms. Extracellular DNA is a crucial component of the bacterially produced EPS constituting the biofilm matrix, with species-dependent roles in cell aggregation and intercellular connection, maintenance of the structure of the biofilm, and as an adhesive with some antimicrobial properties (reviewed by Flemming and Wingender, 2010). Brown et al. (2015) showed that the exogenous addition of DNase I led to rapid degradation of extracellular DNA and removal of a *C. jejuni* biofilm attached to stainless steel (to mimic a food processing environment). *C. jejuni* is capable of both formation of *de novo* biofilms as well as integration into existing biofilms occupied by other species in food related environments (Teh et al., 2014). The use of DNase I in this study against a *C. jejuni* biofilm was successful in both swift removal of the biofilm from its attached surface and in prevention of reattachment and *de novo* synthesis of a new biofilm for up to 48 h on a DNase I treated surface. Kim et al. (2017) showed that DNase I significantly inhibited the biofilm forming capabilities of one *C. jejuni* and three *Campylobacter coli* strains when added at the beginning of biofilm formation and also disrupted 72 h old mature biofilms of these strains, isolated from commercially bought raw chickens. This study further contributes to the assumption that extracellular DNA plays a key role in *Campylobacter* biofilm formation, highlighting DNase I as a promising candidate for the control of *Campylobacter* biofilms. Zetzmann et al. (2015) reported the formation of DNase I-sensitive biofilms by *L. monocytogenes* EGD-e at low ionic strength, conditions which are commonplace in food processing. DNase I was also found to be effective against biofilm formation in a study carried out by Harmsen et al. (2010) in which its employment inhibited initial attachment of *L. monocytogenes* cultures to glass and delayed biofilm formation in polystyrene microtiter plates.

### Bacteriophage

Bacteriophage are bacteria’s natural enemies and so have potential for use against pathogenic and spoilage bacteria in food (reviewed by Endersen et al., 2014). Phage offer special promise when it comes to eradicating biofilms as they are capable of penetrating the matrix and diffusing through the mature biofilm and, once inside, express their antibacterial properties (Briandet et al., 2008; Donlan, 2009), as illustrated in **Figure 2B**. Work has also been carried out against biofilms with both natural and engineered phage (reviewed in Simões et al., 2010). Phage are extremely specific to their bacterial host and this specificity is important for use in control of undesirable bacterial species in foods as beneficial bacteria are often used in food production, especially starter cultures in fermented foods, in which cases the preservation of the beneficial bacteria is essential for finished product quality (Guenther et al., 2009). Lytic phage are better suited to biocontrol purposes as, unlike lysogenic phage, they engage the lytic pathway to the detriment of the bacterial cell. LISTEX^TM^ is a commercial product developed from the bacteriophage P100 which induces cell lysis and disintegration of the EPS by enzymatic action. It is a natural and non-toxic phage product active against *L. monocytogenes* and is recognized in the USA by the United States Department of Agriculture (USDA) for use in all food products (Listex, 2006). Soni and Nannapaneni (2010) treated 21 *L. monocytogenes* strains, which had formed biofilms on stainless steel coupons, with bacteriophage P100 and reported a significant reduction in cell numbers of the listerial biofilms. Lytic phage φ S1 was shown to be effective against early stage biofilms of *P. fluorescens* (Sillankorva et al., 2004). The biofilms were 5 days old when treated with the bacteriophage φ S1 and this resulted in an 80% removal of the biofilm (under optimal conditions). Another study demonstrated the efficacy of phage K plus six derivatives in the removal and prevention of *S. aureus* biofilms in microtitre plates (Kelly et al., 2012). CHAP_K_, a peptidase derived from the phage K, successfully disrupted and eliminated staphylococcal biofilms on microtitre assay plates within 4 h (Fenton et al., 2013). In a study by Lu and Collins (2007), *E. coli*-specific bacteriophage T7 was engineered to express intracellularly a biofilm-degrading enzyme, dispersin B, which targets an adhesin required for biofilm formation by *E. coli* and *Staphylococcus* spp. during infection, so that when added to the culture medium the phage was able to simultaneously attack the bacterial cells in the biofilm (as phage do) and also able to penetrate the biofilm matrix through degradation of EPS. The group demonstrated that the approach involving the engineered phage was markedly more efficient at biofilm disruption than the use of a non-engineered phage. Building on this work, enzymatic phage designed with multiple EPS targets could greater improve efficiency of this technique.

### Bacteriocins

Ribosomally synthesized antimicrobial peptides secreted by bacteria, known as bacteriocins, or the bacteriocin-producing strains themselves, may be added to culture media to impede initial cell adhesion and biofilm formation of certain susceptible bacteria (da Silva and De Martinis, 2013), as illustrated in **Figure 2C**. Nisin, a bacteriocin secreted by *Lactococcus lactis*, is a safe and effective additive for certain food products (Cotter et al., 2005) and a commercialized form, Nisaplin^®^, is produced by Dupont (formerly Danisco). Nisin A, produced by a *L. lactis* UQ2 isolated from Mexican style cheese, was investigated for its activity against *L. monocytogenes* biofilm formation on stainless steel coupons (García-Almendárez et al., 2008). Both *L. lactis* UQ2 cells and a spray-dried crude bacteriocin fermentate (CBF) of *L. lactis* UQ2 were assessed using fluorescent *in situ* hybridization (FISH) with specific labeled probes to distinguish between cells of both cultures. The study found that a combination of lactic acid and nisin A, both produced by *L. lactis* UQ2, was successful in the restriction of *L. monocytogenes* biofilm formation by competitive exclusion indicated by the observation of reduced numbers of *L. monocytogenes* cells on the steel chips incubated in co-culture with *L. lactis* UQ2 compared to the *Listeria*-only control. In a study by Field et al. (2015), a modified nisin variant with enhanced antimicrobial and anti-biofilm activity against the canine pathogen *Staphylococcus pseudintermedius* was shown to be more effective than the original peptide from which it was derived. The bioengineered bacteriocin was capable of both impairing biofilm formation and reducing pre-existing biofilms of *S. pseudintermedius*. *Lactobacillus sakei* is a bacteriocin producing lactic acid bacteria commonly used in the preservation and fermentation of meat products (Champomier-Verges et al., 2001). *L. monocytogenes* biofilm formation in the presence of an *L. sakei* strain (*L. sakei* 1) and of the cell-free supernatant (CFS) of *L. sakei* 1 containing bacteriocin, sakacin 1, was assessed on stainless steel coupons (Winkelströter et al., 2011). A non-bacteriocin producing *L. sakei* strain and its bacteriocin-free CFS were also co-cultured with the *L. monocytogenes* biofilms separately as controls. The bacteriocin-producing strain and its CFS were both efficient in the inhibition of the initial steps of biofilm formation as they were observed to decrease the number of adhered cells present on the stainless steel coupons. However, after 48 h of incubation re-growth of adhered listerial cells was observed in the culture containing the sakacin 1-CFS only and so, inhibitory activity cannot safely be attributed to bacteriocin-production alone. The results are still promising indicating that *L. sakei* and its bacteriocin may be beneficial for the inhibition of early biofilm formation by *L. monocytogenes*. In a similar study, Pérez-Ibarreche et al. (2016) investigated the effect of bacteriocin-producing *L. sakei* strain CRL1862 on biofilms formed by *L. monocytogenes* FBUNT (isolated from artisanal sausages) on industrially relevant stainless steel and polytetrafluoroethylene (PTFE) surfaces. This *L. sakei* strain was found to be effective at biofilm inhibition, leading to the suggestion by the authors of the pre-treatment of food processing equipment with the *Lactobacillus* or its bacteriocin as a potential method of preventing *Listeria* adhesion to the surface concerned.

Many bacteriocins are produced by lactic acid bacteria which are commonly employed as starter cultures for the production of various fermented foods (Buckenhüskes, 1993). In addition to the acclaimed safety profile of LAB for use in food production, their metabolism is known to offer sensory improvements to fermented food products (Leroy and De Vuyst, 2004) and the presence of selected strains may also inhibit the growth of some foodborne spoilage and pathogenic bacteria, making LAB a practical addition to food preparations and processing cycles. In a recent study, recombinant lectin-like proteins that were identified by genome mining of probiotic *Lactobacillus rhamnosus* GG and over-expressed in *E. coli* were found to disrupt biofilms formed by *S.* Typhimurium ATCC14028 on polystyrene pegs (Petrova et al., 2016). Although, the authors carried out this study with clinical applications in mind, employing such proteins or the probiotic strain itself to battle *Salmonella* biofilms in the food industry is a plausible ambition. Woo and Ahn (2013) discussed competitive exclusion in the context of probiotic mediated exclusion and displacement against biofilm formation of *L. monocytogenes* and *S.* Typhimurium. From milk tanks and milking equipment in two traditional Algerian farms, a *Lactobacillus pentosus* strain was isolated that had strong activity against the adhesion of *S. aureus* cells to polystyrene and stainless steel (Ait Ouali et al., 2014). Additionally, this *L. pentosus* LB3F2 (among other LABs isolated) formed biofilms on the industrially relevant surfaces tested, highlighting its potential for use in food processing as a beneficial biofilm former capable of inhibiting *S. aureus* by creating a protective barrier on equipment surfaces and/or *via* competitive exclusion of the pathogen. In the cases of competitive exclusion and beneficial bacteria with barrier functions it must be considered nonetheless that there is potential for the protective strain to develop resistance to the sanitizer/disinfectant used in cleaning protocols and there exists the possibility of transference of the resistant plasmid to the spoilage/pathogenic strain that it is protecting against.

### Naturally Sourced

Extracts from aromatic plants are being investigated as natural agents against bacterial biofilms (Bridier et al., 2015). They are generally regarded as safe (GRAS) and so are compatible with current regulations regarding food production. Examples include: oregano oil, thymol and carvacrol effective against *Staphylococcus* biofilms (Nostro et al., 2007). *Thymus vulgare* essential oil caused a 90% reduction in AHL production (measured by quantifying violacein production in the AI-1 QS indicator strain *Chromobacterium violaceum* CV026) of *P. fluorescens* KM121 in a 72 h old culture (Myszka et al., 2016). These results were confirmed by liquid chromatography mass spectrometry (LC–MS). The essential oil also strongly inhibited cell adhesion to stainless steel, viewed by fluorescence microcopy and inhibition of adhesion quantified by the scale described by Le Thi et al. (2001). The results showed *P. fluorescens* KM121 first degree adhesion to be dominant on the stainless steel coupons, meaning that on 50 randomly selected visual fields only 0–5 bacterial cells were present post-washing. Extracted from *Euodia ruticarpa* (a plant in the Rutaceae family), the compounds evodiamine and rutaecarpine and a quinolinone fraction were found to reduce biofilm formation of *C. jejuni* NCTC 11168 on stainless steel after 24 h or more (Bezek et al., 2016). In a recent study, *B. subtilis* biofilms formed on polystyrene microtitre plates and stainless steel coupons were treated with 1 and 2% solutions of organic acids (citric, malic, and gallic) isolated from natural sources and additionally chlorine for comparison. Akbas and Cag (2016) reported citric acid as being as effective at biofilm inhibition and disruption as the chlorine standard, results which may encourage exploration of organic acids as a potential natural alternative to chemical substances for *Bacillus* biofilm control. Maderova et al. (2016) employed an unusual method for the control of *P. aeruginosa* biofilms in a water environment by utilizing food waste materials as QS signaling molecule adsorbents. These authors were successful in reporting reduced biofilm formation (without consequence to cell viability) through the addition of spent grain. Magnetic modification of promising food materials, including the grain, allowed for their separation and removal from the water environment. Following the success of this study, the addition (and subsequent removal afterward) of food materials spoiled by ‘safe,’ food grade microbes to certain food processing arrangements could be a possible avenue of research for biofilm control in the food industry.

### Quorum Sensing Inhibitors

**FIGURE 3:**
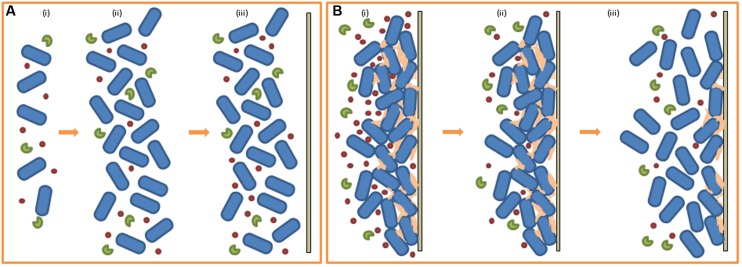
**Quorum quenching (QQ) and biofilm formation. (A)** Effect of QQ molecules on early stage biofilm formation (i) low population density, low QS signal, addition of QQ molecules (ii) high population density, low QS signal, QS molecules degraded by QQs (iii) absence of attachment to solid surface, biofilm formation does not occur. **(B)** Effect of QQ molecules on early pre-existing biofilm (i) biofilm formed, high QS signal, addition of QQ molecules (ii) QS molecules degraded by QQs, reduction of QS signal (iii) decrease in EPS production, release of cells, return of released cells to planktonic state (i.e., reduced biofilm).

Strategies that target quorum sensing and, therefore, biofilm formation (and other virulence factors) as opposed to bactericidal strategies exert less selection pressure to develop resistance to the inhibitory agent. In these instances, bacteria can be ‘controlled’ in place of being killed. Many organisms produce quorum quenching (QQ) molecules when competing with neighboring species for nutrients, space, etc. QQ refers to the inhibition of QS through degradation and/or inactivation of the QS signaling molecules (Dong et al., 2001). The inability of the susceptible bacterial cell to sense and respond to its population density interferes with various secondary cell functions, usually diminishing some aspect of virulence. *P. aeruginosa* metabolizes its own AHL signaling molecules by cleaving QS molecules to form a homoserine or a fatty acid which it consumes as carbon and nitrogen sources (Huang et al., 2003). Signaling molecules are also degraded by the producer to maintain appropriate signal concentration and to prevent improper activation of the QS system. *Agrobacterium tumefaciens* degrades its own QS signaling molecules to terminate QS activities by producing the AHL-lactonase AttM while in its stationary phase of growth (Zhang et al., 2002). The concept of QQ as an anti-biofilm tool lies with the addition of the isolated inhibitory molecule (or the producer itself) as a bioagent in the food industry or its formulation into an antibacterial treatment for clinical use against human pathogens (see **Figure 3**). Strategies employed to prevent biofilm formation targeting the QS system are based on inhibition of cell-to-cell communication, which can be executed in a number of ways, including the inhibition of signaling peptides synthesis or the degradation of the peptides, prevention of signaling peptide–receptor binding or inhibition of the signal transduction cascade further down the line (Brackman and Coenye, 2015). Although a great deal of further study is still required to fully understand the relationship between QS and biofilm formation, it is accepted that QS inhibition is a promising strategy to combat bacterial biofilms. Viana et al. (2009) investigated the role of AHLs in biofilm formation by *H. alvei*, a bacterial food contaminant commonly isolated from raw milk (Ercolini et al., 2009) and cheeses (Coton et al., 2012). Despite *H. alvei* being considered to be an opportunistic human pathogen in some nosocomial infections (Rodríguez-Guardado et al., 2005), the bacterium is often added to certain cheeses to improve taste and aid in ripening and so is considered to be a microorganism with beneficial technological properties for use in food fermentation (Bourdichon et al., 2012). Previous studies (Pinto et al., 2007) have established that *H. alvei* is a producer of AHLs and so the group set out to detect the presence of AHLs in a *H. alvei* biofilm with the objective of establishing a link between QS and biofilm formation. On verifying the presence of AHLs in the biofilm, they also demonstrated the inhibition of biofilm formation by synthetic furanones (previously shown by Manefield et al., 2002). It was also established that *H. alvei halI*, an AHL-synthase gene mutant, was deficient in proper biofilm formation, further strengthening the hypothesis that AHL-mediated QS plays a role in biofilm formation by *H. alvei*. In a study carried out by Van Houdt et al. (2004) *in vitro* biofilm formation was characterized in 68 Gram-negative bacterial strains isolated from a raw vegetable processing line. Accompanying assays using reporter bacteria detected the presence of QS signals produced by each strain. Although, five isolates were determined to produce AHLs and AI-2 signals and a further 26 strains were AI-2 producers, a general correlation between the QS signals detected and measurable biofilm formation was not clear for the strains under investigation. Nevertheless, the authors stipulated that the absence of a link between QS and biofilm formation in their study does not dismiss the influence of signaling molecules in other biofilm formers. Another study highlighted the link between QS and biofilm formation in reporting that *P. aeruginosa lasI* mutant strains that were unable to synthesize the AHL 3-oxo-C12-HSL formed atypical biofilms when cultured in a flow cell (Bjarnsholt et al., 2010). The antibiotic azithromycin was used successfully as a QS blocking agent against the AHLs C4-HSL and 3-oxo-C12-HSL in *P. aeruginosa* and in doing so impacted bacterial biofilm formation by reducing cell adhesion to polystyrene surfaces (Favre-Bonte et al., 2003). Tan et al. (2014) carried out a long term study investigating the role of QS signaling molecules in multi-species microbial communities undergoing granulation through incubation of a mixed bacterial culture in a bioreactor used for water treatment. Simultaneously, they assessed the concentration levels of AHL molecules present at different stages of granule formation. The group found that AHL concentration positively correlated with the behavioral steps involved in granulation and that addition of exogenous AHLs to the culture resulted in increased EPS production, suggesting a role for QS signaling in bacterial granule formation. A later study performed by the same group (Tan et al., 2015) demonstrated that QQ was the primary mode (as opposed to environmental factors) of QS signal reduction and served as a key player in the regulation of different stages of bacterial granulation formation.

Due to the apparent benefits of inhibiting QS, studies screening large libraries/collections of microorganisms in the search for QQ molecule producers have recently emerged. Christiaen et al. (2011) employed a high-throughput approach to screening environmental samples cultivated in minimal media supplemented with AHLs as their sole sources of carbon and nitrogen. These enriched isolates were screened using the QS inhibition selector biosensor strain *P. aeruginosa* QSIS2 (assay developed by Rasmussen et al., 2005), which revealed 41 isolates with QQ activity (in some cases resistant to heat and proteinase K treatments). Kusari et al. (2014) showed that environmentally derived samples of the endophytic bacteria of the plant *Cannabis satvia* L. were capable of quenching four different AHL molecules of the biosensor strain *C. violaceum* which regulates production of the purple pigment violacein through QS signaling activity. Large numbers of diverse unculturable bacteria from environmental samples may also be efficiently screened for QQ activity through the construction and scanning of metagenomic libraries (Coughlan et al., 2015). For example a functional metagenomic library assembled from soil samples was screened using a QQ biosensor assay employing *A. tumefaciens* NTL4 as an indicator microorganism and in doing so identified three active clones (including two novel lactonases) capable of reducing motility and biofilm formation in *P. aeruginosa* (Schipper et al., 2009). Studies describing the identification of quorum quenching molecules are briefly summarized in **Table 1**.

**Table 1:** Studies describing quorum quenching molecules.

QQ molecule/activity	Producing spp./closest known relatives	Environment sampled	Attenuated virulence of:	Reference
Plant extracts	*Conocarpus erectus, Chamaesyce hypericifolia, Callistemon viminalis, Bucida buceras, Tetrazygia bicolor*, and *Quercus virginiana*	Extracts of six South Florida plants	*Pseudomonas aeruginosa* PAO1 (biofilm reduction)	Adonizio et al., 2008
Novel oxidoreductase	*Acidobacterium* spp. MP5ACTX8	Functional metagenomic library, soil, University of Göttingen, Germany	*P. aeruginosa* PAO1	Bijtenhoorn et al., 2011
Broad spectrum lactonase activity	Genera *Acinetobacter*, *Klebsiella*, and *Burkholderia*	Rhizosphere of ginger (*Zingiber officinale*), Rimba Ilmu, University of Malaya, Malaysia	*P. aeruginosa* PAO1, *Erwinia carotovora* strain GS101 and PNP22	Chan et al., 2011
Lactonase (AiiA_B546_ expressed in *Pichia pastoris* GS115)	*Bacillus* spp. B546	Mud of a fish Pond, Wuqing, Tianjin, China	*Aeromonas hydrophila* ATCC 7966	Chen et al., 2010
41 strains utilizing AHLs as carbon/nitrogen source. 14 with extracellular QQ activity	21 genera, most common *Pseudomonas*, *Arthrobacter*, and *Aeromonas*	16 soil and water samples	N/A	Christiaen et al., 2011
Lactonase (encoded by *aiiA*)	*Bacillus* spp. 240B	Soil	*E. carotovora* strain SCG1	Dong et al., 2000
Furocoumarins, grapefruit juice	N/A	Grapefruit and grapefruit juice	*Escherichia coli* O157:H7, *Salmonella* Typhimurium and *P. aeruginosa* (biofilm reduction in all three)	Girennavar et al., 2008
Acylase Lactonase	*P. aeruginosa* PAO1 *Pseudomonas* strain PAI-A	N/A Soil	Both utilized own AHLs as carbon/nitrogen sources	Huang et al., 2003
Lactonase	*Rhizobium* spp. strain NGR234	N/A	*P. aeruginosa* PAO1 (including biofilm reduction)	Krysciak et al., 2011
Acylase (*aiiD* expressed in *P. aeruginosa)*	*Ralstonia* strain XJ12B	Biofilm in experimental water treatment system, The National University of Singapore	*P. aeruginosa* PAO1	Lin et al., 2003
Lactonase (AidH)	*Ochrobactrum* spp.	Soil, Yunnan Province, China	*Pectobacterium carotovorum* Z3-3, *Pseudomonas fluorescens* 2P24 (biofilm reduction)	Mei et al., 2010
Acylase (*aac* expressed in *E. coli* and *Shewanella oneidensis*)	*Shewanella* spp. strain MIB015	N/A	*Vibrio anguillarum* TB0008 (biofilm reduction)	Morohoshi et al., 2008
Lactonase (*aiiM)*	*Microbacterium testaceum* StLB037	Leaf surface of the potato	N/A (identified through genome sequencing)	Morohoshi et al., 2011
Lactonase (*ahlS*)	*Solibacillus silvestris* StLB046	Leaf surface of the potato	*P. carotovorum* subsp. *carotovorum*	Morohoshi et al., 2012
Essential oils thymol, carvacrol, eugenol	N/A	Sigma-Aldrich Chemicals (St. Louis, MO, USA)	Effective against paper mill-associated biofilms	Neyret et al., 2014
Essential oils oregano (*Origanum vulgare* L.) oil, carvacrol, thymol	*Origanum vulgare* L. N/A N/A	Ocular infections	*Staphylococcus aureus, Staphylococcus epidermidis* (biofilm reduction)	Nostro et al., 2007
Two novel lactonases, one known lactonase	*Nitrobacter* spp. Strain Nb-311A, *P. fluorescens, Xanthomonas campestris*	Soil functional metagenomic library	*P. aeruginosa* (biofilm reduction)	Schipper et al., 2009
Acylase (PA2385)	Purified from *P. aeruginosa* PAO1	Holloway collection	*P. aeruginosa* PAO1	Sio et al., 2006
Two lactonases	*Phialocephala fortinii*, *Ascomycete* isolate, *Meliniomyces variabilis* and a potential mycorrhizal isolate	16 isolates of mycorrhizal and non-mycorrhizal root-associated fungi	N/A	Uroz and Heinonsalo, 2008
Amidolytic activity	*Comamonas* spp. strain D1	Soil	*P. carotovorum* strain GS101	Uroz et al., 2007
Novel lactonase (*qsdA*)	*Rhodococcus erythropolis* strain W2	N/A	N/A	Uroz et al., 2008
Acylase (PA0305 expressed in *E. coli* and *P. aeruginosa)*	Purified from *P. aeruginosa* PAO1	N/A	*P. aeruginosa* PAO1	Wahjudi et al., 2011
Novel lactonase (AiiM protein)	*M. testaceum* StLB037	Leaf surface of the potato	*P. carotovorum* subsp. *carotovorum*	Wang et al., 2010
Acylase activity	*P. aeruginosa* strain MW3A	Subsurface seawater, Malacca, Malaysia	N/A	Wong et al., 2012a
Acylase activity	*P. aeruginosa* strain 2SW8	Tropical wetland water, Malaysia	N/A	Wong et al., 2012b
Lactonase-like paraoxonase	N/A	Serum of six mammalian spp.	Hydrolysis of *P. aeruginosa*-specific AHLS	Yang et al., 2005
Lactonase (*aiiA* expressed in *E. coli*)	*Bacillus amyloliquefaciens* strain PEBA20	Laboratory collection strain	*P. carotovorum* subsp. *carotovorum*	Yin et al., 2010

Quorum quenching activity is predominantly due to the action of certain enzymes that degrade QS molecules such as AHLs. It is thought that there are four potential cleavage sites in AHL QS molecules for cutting by enzymes (Chen et al., 2013). Two microbial enzyme families exist that are capable of cleaving AHL structures. Class I includes lactonases, acylases and paraoxonases. Lactonases or decarboxylases catalyze the degradation of the homoserine lactone ring. Dong et al. (2000) initially reported the AHL-degrading activity of a lactonase encoded by a gene (*aiiA*) cloned from *Bacillus* spp. 240B through cleavage of the lactone ring from the acyl moiety, which inhibited virulent activity of the plant pathogen *Erwinia carotovora*. The AidH AHL-lactonase from *Ochrobactrum* spp., which hydrolyzes the ester bond of the homoserine lactone ring of AHLs, has a very broad range of targets and is effective at reducing biofilm formation of the food spoilage bacterial strain *P. fluorescens* 2P24 (Mei et al., 2010). AiiA_B546_ AHL-lactonase from *Bacillus* spp. B546 displayed a broad range of AHL substrate specificity and showed promise for use in reducing fish mortality by controlling the pathogen *Aeromonas hydrophila* (Chen et al., 2010). Cao et al. (2012) reported the oral administration of a broad-spectrum, thermostable and protease resistant AiiA_AI96_ AHL-lactonase from *Bacillus* spp. AI96 to be successful in the attenuation of *A. hydrophila* infection in zebrafish. In another study, three bacterial strains with QQ activity were isolated from the rhizosphere of ginger (*Zingiber officinale*) from the Malaysian rainforest. The strains belonging to the genera *Acinetobacter* and *Klebsiella* possessed broad spectrum lactonase activity while the *Burkholderia* strain was capable of reduction of 3-oxo-AHLs to 3-hydroxy compounds, thus inactivating the AHL signaling molecules. All three strains were found to attenuate virulence of *P. aeruginosa* and *E. carotovora* in co-culture assays (Chan et al., 2011).

Acylases or deaminases cleave an AHL into a homoserine lactone ring and a free fatty acid moiety through hydrolysis of their amide link (Lin et al., 2003). AHL-acylases generally show higher substrate specificity than lactonases for AHL molecules based on the length of their acyl side chains. AHL-acylase AiiD has a higher affiliation for the degradation of long chain AHLs. Cloning of the *aiiD* gene from *Ralstonia* strain XJ12B into *P. aeruginosa* resulted in inhibition of AHL 3-oxo-C10-HSL accumulation and interference with some QS related traits (Lin et al., 2003). Genes encoding acylases capable of degrading the primary QS signaling molecules of *P. aeruginosa* exist within the *P. aeruginosa* PAO1 genome itself. *quiP* and *pvdQ* encode acylases which specifically degrade 3-oxo-C12-HSL and AHLs with long acyl chains only, excluding those with short acyl chains (Sio et al., 2006). An additional AHL acylase in the *P. aeruginosa* PAO1 genome was reported by Wahjudi et al. (2011). The *pa0305* gene, predicted to encode a penicillin acylase, was cloned and its functional protein PA0305 characterized. The protein was shown to degrade AHLs with acyl side chains of 6–14 carbons in length and its overexpression reduced both accumulation of the QS signaling molecule 3-oxo-C12-HSL and virulence of *P. aeruginosa*. Morohoshi et al. (2008) showed that expression of the *aac* gene from *Shewanella* spp. strain MIB015 in the fish pathogen *Vibrio anguillarum*, which is known to produce three distinct AHL signaling molecules and to regulate biofilm formation through QS (Croxatto et al., 2002), resulted in reduced biofilm formation on a polypropylene plastic surface. An AHL-degrading bacterial strain was isolated from a sea water sample collected in Malacca, Malaysia (Wong et al., 2012a). This strain, which contained genes with high homology to known acylases, was capable of utilizing *N*-(3-oxohexanoyl)-L-homoserine lactone as its sole carbon source and degrading AHLs with and without 3-oxo group substitution at the C3 position in the acyl side chain. The strain was also observed to release AHLs (detected in the supernatant) indicating both QS and QQ activity. This group also isolated a strain with similar activity and phylogenetic roots from tropical wetland water also in Malacca (Wong et al., 2012b).

Another type of QQ enzyme is the lactonase-like paraoxonases isolated from mammalian sera. Enzymes isolated from mammalian sera were reported to be capable of hydrolyzing the lactone ring of AHLs produced by *P. aeruginosa* (Yang et al., 2005). Other examples of anti-QS agents isolated from eukaryotes include two lactonases isolated from a collection of root-associated fungi (Uroz and Heinonsalo, 2008) and various quorum quenchers derived from plants (reviewed by Koh et al., 2013). Class II microbial AHL-targeting enzymes are oxidoreductases which target the acyl side chain of AHL molecules and catalyze a modification of the chemical structure of the signal, that is not degraded (Chen et al., 2013). A novel oxidoreductase identified from a metagenomic library reduced pyocyanin production, motility and biofilm formation when expressed in *P. aeruginosa* PAO1 (Bijtenhoorn et al., 2011).

AI-2 QS signaling systems may also be potential anti-biofilm targets. As previously mentioned, *luxS* influences biofilm formation in *L. monocytogenes* (Sela et al., 2006). Potential blockers of AI-2 signal synthesis have been investigated by Zhao et al. (2003) and Alfaro et al. (2004) with the successful design of synthetic AI-2 inhibitors reported by Shen et al. (2006) that act as competitive inhibitors of the LuxS protein interfering with the synthesis of AI-2 precursors. Recently, from a functional metagenomic library, Weiland-Bräuer et al. (2016) reported the identification of a clone originating from a German Salt Marsh to be effective at prevention of biofilm formation in *Klebsiella oxytoca* M5a1 and *K. pneumonia*e isolated from patients with urinary tract infections, species with reported AI-2 mediated QS (Balestrino et al., 2005; Zhu et al., 2011). The purified protein was suspected to possess oxidoreductase activity. To date, the AIP system in Gram-positive bacteria has not been examined as a target for potential biofilm inhibition but it may prove to be a promising route for future study.

As discussed above, the isolation of anti-biofilm agents from nature is an attractive prospect, leading to the search for quorum quenchers from organic sources. Girennavar et al. (2008) reported QS inhibition in *V. harveyi* biosensor strain by grapefruit juice and bioactive extracts from grapefruits. Additionally, they were also found to be capable of inhibition of biofilm formation by *E. coli* O157:H7, *S.* Typhimurium and *P. aeruginosa*, species which often prove troublesome for the food industry. In another study, extracts from six South Florida plants were effective in impacting QS signaling in *P. aeruginosa* with significantly reduced biofilm formation observed in the presence of extracts from three of these plants (Adonizio et al., 2008).

## Conclusion and Future Prospects/Directions

The majority of bacteria, including those detected in food processing environments, are gifted with the ability to resist standard cleaning measures by their capacity to form biofilms on many of the surfaces approved for use in the food industry. This persistence leads to increased microbial load in both the food processing environments and in the subsequent food products, leading to food spoilage and reduced shelf life and also to increased risk of infectious outbreaks originating from food sources. Food safety is a global concern and increased risk of infection is accompanied by a requirement for more stringent and frequent evaluation of food manufacture and processing plants. Economic losses suffered by food production facilities and health related costs faced during foodborne pathogen epidemics mean that the presence of biofilm-forming bacteria can have a considerable impact on food processing establishments and, so, impeding their ability to persist in these environments is a very attractive objective for both food industry workers and researchers.

Current strategies show promise in laboratory-based experiments, with the successful inhibition of biofilm formation reported in numerous studies. However, there are considerations when applying these approaches to real life situations that limit their value to the food industry. Firstly, it is important that anti-biofilm agents used in food processing facilities meet safety requirements outlined by appropriate regulatory bodies. Agents deemed successful in the lab must also be tested and proven safe for application to food contact surfaces and, especially, if such agents are to be added to the food product itself. Ideally, quorum quenchers derived from food-grade microorganisms, plants and other natural sources would be most suitable. Additionally, researchers developing anti-biofilm strategies must acknowledge that product quality is a top priority for food manufacturers, and so, biofilm inhibitors must not influence the taste, texture or palatability of the food in any way. This is especially relevant to the dairy industry, where many fermented milk products are developed using specific populations of microorganisms in a carefully refined system that is sensitive to change. Here, strategies that target QS signaling over growth inhibitors or bactericidal agents are useful as they do not threaten the lives of useful bacteria in the process. In such cases, the specificity of the quorum quencher is significant so as not to inhibit QS signals of beneficial bacteria that may regulate certain factors responsible for their fermentation abilities and perhaps the production of particular by-products that lend aromas and textures to the finished food. Searching for quorum quenchers from the food processing environment itself may prove useful here as competition among microbes occupying the same niche leads to the production of compounds, such as bacteriocins and QS inhibitors, specific to their common competitors. This approach may increase the likelihood of discovering quorum quenchers with action specific against the target bacteria. Another necessary factor to consider when introducing a lab-derived method to an industrial setting is the practicality of the biofilm-fighting strategy proposed. Notably with QS inhibitors, being derived from living organisms and often vulnerable to harsh climates, the active bioagents must be capable of withstanding conditions typical of food processing environments. Heat stability as well as activity at low temperatures, a broad pH range of action and resistance to proteases are all attractive qualities in a food-grade quorum quencher, depending on the process in question.

Quorum quenching has been shown to be a promising avenue of anti-biofilm research in food microbiology, with limitations faced in the transferal of laboratory findings to industrial applications. As discussed above, the criteria outlining a suitable QS inhibitor for inhibition of biofilm in the food industry is a detailed and extensive list. The search continues, employing a number of screening techniques on samples from exotic and domestic sources alike.

## Author Contributions

LC, PC, CH, and AA-O designed the manuscript; LC and AA-O wrote the manuscript; PC and CH critically revised the manuscript.

## Conflict of Interest Statement

The authors declare that the research was conducted in the absence of any commercial or financial relationships that could be construed as a potential conflict of interest.
